# Abiotic Factors Promote Cell Penetrating Peptide Permeability in Enterobacteriaceae Models

**DOI:** 10.3389/fmicb.2019.02534

**Published:** 2019-11-26

**Authors:** Daichi Toyohara, Yasuhito Yokoi, Go Inoue, Takahiro Muraoka, Tetsushi Mori

**Affiliations:** ^1^Department of Biotechnology and Life Science, Tokyo University of Agriculture and Technology, Koganei, Japan; ^2^Department of Organic and Polymer Materials Chemistry, Tokyo University of Agriculture and Technology, Koganei, Japan

**Keywords:** cell penetrating peptides, CPP permeation, abiotic factors, temperature, solution tonicity

## Abstract

Conventionally, the delivery of biomolecules into bacteria for the generation of characterized or functional mutants has relied greatly on horizontal gene transfer techniques. However, the low compatibility of these techniques with novel or hard-to-transform bacteria currently serves as a challenge to the bioengineering field. Here, we explored the use of cell penetrating peptides (CPPs) as an alternative biomolecule delivery approach by investigating the effects of the abiotic factors during CPP permeation. Using the (KFF)_3_K-FAM conjugate and *Escherichia coli* as models, we evaluated four abiotic factors where two of these factors, temperature and solution tonicity, promoted (KFF)_3_K-FAM permeation efficiency. Our data show that optimal (KFF)_3_K-FAM permeation efficiency was achieved for *E. coli* at approximately 98.1% under conditions of 37°C (growth optimal temperature) and 50% PBS concentration. Based on these conditions, we subsequently tested the applicability of CPP permeation in various bacterial strains by treating 10 bacterial strains from the Enterobacteriaceae family among which seven strains have no CPP permeation records with (KFF)_3_K-FAM. Interestingly, when compared with non-optimized conditions, all 10 strains showed a marked increase in CPP permeation ranging between 20 and 90% efficiency. Although using strains within Enterobacteriaceae that are phylogenetically close, our results hinted on the possibility that with proper optimization of the abiotic factors, CPPs could be compatible with a broad range of bacterial strains. Our efforts suggest that CPP could serve as an effective alternative approach for mutant generation and for biomolecule delivery into novel or hard-to-transform bacteria.

## Introduction

In bacterial genetic engineering, the success of generating characterized or functional mutants is pivotal for the advancement of the field. To generate such mutants, techniques for biomolecule delivery and its efficacy are crucial factors. Thus far, the delivery of biomolecules, mainly DNA, into bacteria have relied greatly on horizontal gene transfer techniques such as electroporation (Kotnik et al., 2015), conjugation (Goessweiner-Mohr et al., 2014; Cabezon et al., 2015), and transduction (Yosef et al., 2017). Once optimized for the target bacteria, these techniques can be easily performed to generate the necessary genetic mutants. However, the application of these techniques with newly discovered strains has often been a debate since the initial optimization steps are usually tedious and time consuming (Aune and Aachmann, 2010; Wang et al., 2015). Furthermore, it is also known that several cultivable strains are hard to transform with these current techniques (Monk et al., 2012). Recently, efforts to establish delivery systems that are simpler, efficient, and potentially applicable to both novel and hard-to-transform strains using nanoparticles (Deshmukh et al., 2019; Gonzalez-Paredes et al., 2019), organic molecules (Giedyk et al., 2019), and transposable elements (Liu et al., 2018) as carriers have been introduced. Nevertheless, many of these systems currently face challenges in terms of cytotoxicity toward the recipient host, applicability of the system with diverse bacterial strains and low delivery efficiency.

Among these newly introduced approaches, cell penetrating peptides (CPPs) have been in the center of attention as a potential alternative approach (Lindgren et al., 2000; Guidotti et al., 2017; Borrelli et al., 2018). CPPs are short peptides of 30 amino acid residues or less with the ability to permeate through lipid bilayer membranes without the aid of membrane-related transporters or proteins (Avci et al., 2018). Given this advantage, CPPs have been employed as carriers to numerous biomolecules including oligonucleotides, proteins, and chemical compounds in both eukaryotic (Ramsey and Flynn, 2015; Kristensen et al., 2016) and prokaryotic systems (Rajarao et al., 2002; Gomarasca et al., 2017). Thus far, applications of CPPs with bacteria are still limited and have been mainly directed toward the eradication of pathogenic or antibiotic-resistant strains where they are conjugated with gene-specific antisense oligonucleotides (ASOs) that directly suppress the transcription of target genes intracellularly (Xue et al., 2018).

In our work, we hope to employ CPPs as carriers to deliver biomolecules into bacteria to promote the development of an efficient mutagenesis technique applicable with novel and hard-to-transform strains. In order to develop a highly efficient and robust delivery system, several major issues needed to be addressed. The first would be to determine the factors that may affect or influence CPP permeation; the second, to evaluate the compatibility of CPPs and the flexibility of its application with a broad range of bacterial genera; the third, to design plasmids or linear DNA fragments applicable for efficient mutant generation upon intracellular transfer *in vivo*; and finally, to develop and optimize techniques to efficiently synthesize CPP-plasmid/linear DNA conjugates applicable with bacterial systems. Here, addressing the first two issues, we used the [KFFKFFKFFK; (KFF)_3_K] CPP as a model and reported on the identification of factors influencing CPP permeation by focusing on the abiotic factors employed in CPP permeation assays. Upon optimization, we subsequently demonstrated the applicability of these factors to promote CPP permeation in several Enterobacteriaceae model strains including those that have not been CPP tested.

## Materials and Methods

### Bacterial Strain and Culture Conditions

*Escherichia coli* K-12 (ATCC10798) was purchased from the American Type Culture Collection (ATCC; Virginia, USA). *Citrobacter werkmanii* (NBRC105721), *Edwardsiella ictaluri* (NBRC105724), *Enterobacter hormaechei* (NBRC105718), *Erwinia persicina* (NBRC102418), *Pantoea agglomerans* (NBRC102470), *Pectobacterium carotovorum* (NBRC14082), *Proteus hauseri* (NBRC3851), *Serratia grimesii* (NBRC13537), *Trabulsiella guamensis* (NBRC103172), and *Yersenia bercovieri* (NBRC105717) were purchased from the NITE Biological Resource Center (NBRC; Chiba, Japan). All bacterial strains were selected and handled based on the biosafety guidelines provided by our institution. *E. coli* was cultured at 37°C, *E. persicina* at 25°C while all other strains were cultured at 30°C with agitation. Luria Bertani (LB; 10 g/L Tryptone, 5 g/L yeast extract, 10 g/L NaCl, pH 7.0) broth was used as the culture medium. All experiments were performed with cells revived from cryostorage cultivated overnight for 16–20 h. Cells with an average OD_600_ of 1.0 measured with a Mini photo 518R (Taitec Inc., Tokyo, Japan) were used for subsequent experiments.

### (KFF)_3_K-FAM Conjugate

The (KFF)_3_K CPP sequence used in this work was derived from a previous report (Good et al., 2001). (KFF)_3_K conjugated with the 5,6-carboxyfluorescein (FAM) fluorophore [(KFF)_3_K-FAM] was synthesized by greiner bio-one (Tokyo, Japan) based on the Fmoc solid-phase peptide synthesis method. The synthesis yield was 1.97 mg with purity at 97.65% and observed mass signal was at 1772.099. Upon arrival, (KFF)_3_K-FAM was dissolved in *N*,*N*-dimethylformamide (DMF; Sigma-Aldrich, Tokyo, Japan), prepared as a 100-μM stock solution and stored as 5- to 10-μl single use aliquots at −20°C. Working solutions were prepared by diluting the stock solution to the designated concentrations with Phosphate-Buffered Saline (PBS, pH 7.4; Thermo Fisher Scientific, Tokyo, Japan).

### (KFF)_3_K-FAM Permeation Assay

A total of 100 μl of bacterial cells was collected from a 5-ml overnight culture by centrifugation at 7,500× g, 10 min. The collected cells were subsequently washed with 100 μl of PBS three times with centrifugation conditions at 7,500× g, 5 min. Upon washing, the cells were suspended in 50 μl of a given concentration of (KFF)_3_K-FAM and incubated for 1 h. After incubation, the (KFF)_3_K-FAM treated cells were washed three times with PBS to remove excessive (KFF)_3_K-FAM. The whole assay, including centrifugation and incubation, was performed at room temperature (RT, 23°C).

### Optimization of (KFF)_3_K-FAM Permeation Assay Conditions

The optimization of (KFF)_3_K-FAM permeation was performed based on four abiotic factors, which were temperature, incubation time, (KFF)_3_K-FAM concentration, and PBS tonicity. *E. coli* was used as the model bacteria. Optimization steps were performed sequentially and as follows: For temperature, cells were treated with 2 μM of (KFF)_3_K-FAM for 1 h, at 4, 23°C (RT), 30, 35, 40, 45, and 50°C, respectively. For incubation time, *E. coli* was treated with 2 μM of (KFF)_3_K-FAM at 37°C and permeability was evaluated at 5, 15, 30, 45, 60, 90, and 120 min. For (KFF)_3_K-FAM concentration, (KFF)_3_K-FAM was varied from 0.2, 0.5, 1.0, 2.0, 4.0, and 10.0 μM with incubation conditions at 37°C for 1 h. Finally, for PBS tonicity, 100, 75, 50, and 25% PBS were prepared. *E. coli* and 2 μM (KFF)_3_K-FAM were incubated in these solutions at 37°C for 1 h. As a control, a sample incubated in ultrapure water (H_2_O) was also prepared. The negative controls for all the optimization steps were (KFF)_3_K-FAM non-treated cells incubated at RT for 1 h. All samples were performed in triplicates and incubation was performed in a C-1000 Touch Thermal Cycler (BioRad, Tokyo, Japan).

### Generation of *E. coli* Spheroplasts and Cell Penetrating Peptide Permeation Evaluation

Spheroplasts were generated based on the giant spheroplasts preparation protocol without the formation of long filamentous cells (Sun et al., 2014). Briefly, 200 μl of bacterial cells was collected from a 5-ml overnight culture, washed once with PBS, and pelleted as described above. The cell pellet was resuspended in 500 μl of 800 mM sucrose solution and reagents for spheroplast formation were added in the following order: 30 μl 1 M Tris–HCl (pH 8.0), 24 μl 0.5 mg/ml lysozyme, 2 μl 5 U/μl DNase, and 125 mM EDTA-NaOH (pH 8.0). After a 20-min incubation at RT, 100 μl of STOP solution [10 mM Tris–HCl (pH 8.0), 700 mM sucrose, 20 mM MgCl_2_] was added to stabilize the generated spheroplasts. Successful spheroplast formation was confirmed by microscopy. The generated spheroplasts were immediately used for the evaluation of (KFF)_3_K-FAM permeability. Here, incubation was performed with 2 μM (KFF)_3_K-FAM, at 37°C for 1 h.

### Bacterial Staining

The bacterial staining dyes Trypan Blue (TB; Sigma-Aldrich, Tokyo, Japan), SYTO 9 (Thermo Fisher Scientific, Tokyo, Japan), and FM 4–64 (Thermo Fisher Scientific, Tokyo, Japan) were used in this work. Prior to TB staining, 0.4% TB was diluted to 0.1%. (KFF)_3_K-FAM permeated cells prepared as described above were washed three times with PBS, pelleted and resuspended in 50 μl 0.1% TB. For analysis of TB stained cells with flow cytometry, stained cells were washed once with PBS to remove excessive TB. FM 4–64 and SYTO 9 staining was performed based on the manufacturer’s protocol. Observation of both TB and FM4–64 stained cells with fluorescence microscopy was performed immediately upon resuspension. For SYTO 9 stained cells, cells were washed three times with PBS prior to microscopic observation.

### Fluorescence Microscopy

All bacterial samples were observed, and images were captured with a BX53 upright fluorescence microscope equipped with a DP74 digital camera and a 100× objective lens (Olympus, Tokyo, Japan). (KFF)_3_K-FAM permeated cells and dye stained cells were individually observed using the U-FBWA and U-FGW mirror units for green and red fluorescing cells, respectively. Simultaneous observation of green and red fluorescing cells was performed using the U-FBW wideband mirror unit. For direct image comparison among samples, all images were captured using identical exposure times.

### Flow Cytometric Analysis and Cell Sorting

All bacterial samples were detected and sorted using a BD FACSAria™ II cell sorter equipped with a 488-nm blue laser (BD Biosciences, Tokyo, Japan). Green and red fluorescing cells were detected using the 530/30-nm FITC and 710/50-nm PerCP-Cy5.5 filters, respectively. Fluorescence compensation and data analysis were performed using the BD FACSDiva™ software provided with the cell sorter system. Thresholds to determine fluorescing cells was set based on (KFF)_3_K-FAM non-treated cells and the permeation efficiency was presented as the number of fluorescing cells above a given threshold. The evaluation of live-dead cells was performed by sorting cells using the “single-cell” feature onto LB agar plates at 240 colonies per plate. Colony forming units were counted and the percentage of live-dead cells was determined by comparison with sorted non-(KFF)_3_K-FAM treated cells.

### Image Editing and Data Presentation

Microscopic image editing was kept to a minimum and images were cropped to the required sizes using Adobe Photoshop CC (Adobe Systems, Tokyo, Japan). All graphs including *t*-test scores were generated using Prism8 (GraphPad Software, California, USA).

## Results

### Fluorescence Microscopy Validates (KFF)_3_K-FAM Permeation

**Figure 1:**
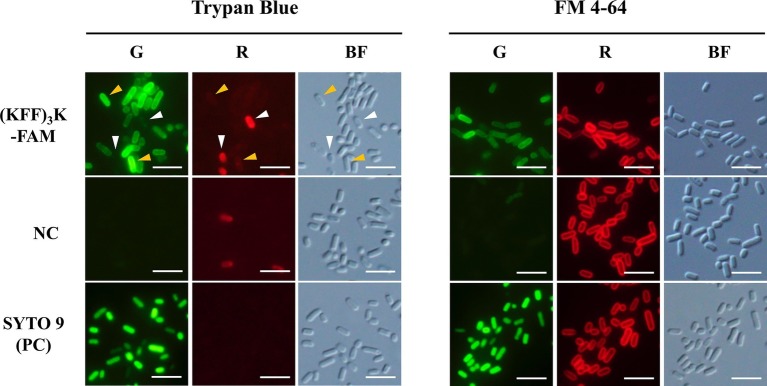
Evaluation of (KFF)_3_K-FAM permeation into *E. coli* cells with Trypan Blue (TB), FM 4–64, and SYTO 9. (KFF)_3_K-FAM permeation was performed at 2 μM concentration in PBS at room temperature (23°C) for 1 h. Untreated *E. coli* cells and SYTO 9 treated cells were used as the negative (NC) and positive controls (PC), respectively. Staining of TB and FM 4–64 was performed immediately after (KFF)_3_K-FAM incubation and fluorescence microscopy images were captured using the green (G) and red (R) fluorescence channels. Cell integrity was confirmed by bright field (BF) images. Images of (KFF)_3_K-FAM permeated cells and the negative controls were captured at similar exposure times to allow for comparison. Yellow arrows indicate (KFF)_3_K-FAM permeated cells (alive), and white arrows indicate TB stained cells (dead). Scale bar is 5 μm.

To ensure that our subsequent analyses were based on CPP permeated cells, we first validated the permeation and localization of (KFF)_3_K-FAM within *E. coli via* fluorescence microscopy. For permeability validation, TB was used. TB emits red fluorescence when excited with blue or green light and cells with an intact membrane are impermeable to the compound (Harrisson et al., 1981). It is also used as a green fluorescence quencher to validate the internalization of biomolecules (Benincasa et al., 2009). Upon incubation of *E. coli* with 2.0 μM (KFF)_3_K-FAM and 0.1% TB, we observed both green and red fluorescing cells with the former being the major population (Figure 1; left panel). Based on this observation and with reference to the negative and positive controls, we confirmed that the green fluorescing cells were alive and (KFF)_3_K-FAM permeated (yellow arrows). The red fluorescing cells (white arrows) on the other hand, were either dead or membrane disrupted. Quantitative analysis using flow cytometry showed that the average (KFF)_3_K-FAM permeation efficiency was 48.1 ± 7.3% with the dead cell population being 1.7 ± 0.7% (Supplementary Figure S1; before optimization).

**Figure 2:**
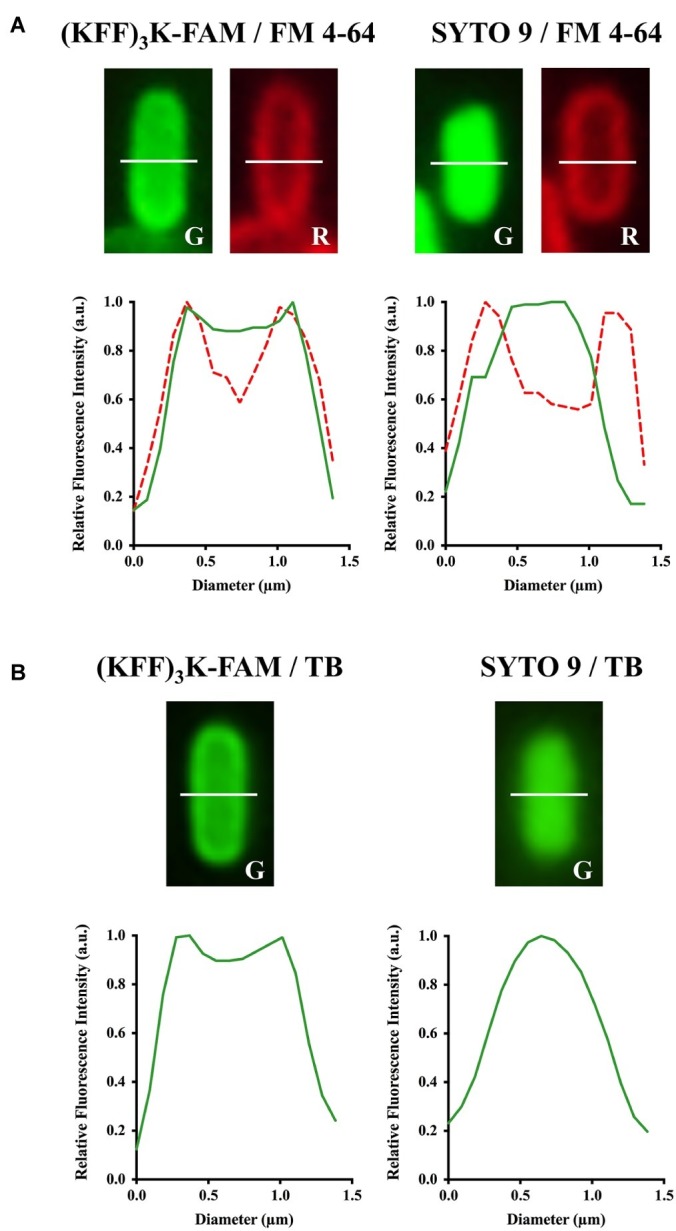
Confirmation of (KFF)_3_K-FAM permeation into the cytoplasm of *E. coli*. (KFF)_3_K-FAM or SYTO 9 treated cells were stained with **(A)** FM 4–64 or **(B)** Trypan Blue (TB). (KFF)_3_K-FAM permeation and dye staining are as performed in Figure 1. *E. coli* cells stained with SYTO 9 and either FM 4–64 or TB were used as controls. Single cells were randomly selected from fluorescence microscopic images and fluorescence values from the cross section (white dotted line) of the respective staining combinations were extracted to provide quantitative representations of internalized (KFF)_3_K-FAM. The fluorescence values for TB stained cells in the red fluorescence channel were equivalent to the background, thus excluded from the current analysis.

Next, to validate the localization of (KFF)_3_K-FAM upon permeation, FM 4–64 and SYTO 9 were used. FM 4–64 specifically stains the cellular membrane while SYTO 9 is a membrane-permeable nucleic acid stain. Both dyes were used in combination with (KFF)_3_K-FAM to determine its localization. As shown, all samples when observed in the red channel showed fluorescing cells with only their cellular membrane stained (Figure 1; right panel). However, when the samples were observed in the green channel, only the (KFF)_3_K-FAM and SYTO 9 (positive control) treated cells showed green fluorescence. Furthermore, all the cells were observed to fluoresce right up to their interior, suggesting that (KFF)_3_K-FAM was internalized into the cytoplasm of the cells. To further validate this, single-cell images from the (KFF)_3_K-FAM/FM 4–64 and SYTO 9/FM 4–64 treated cells were extracted and fluorescence intensity of both the red and green channels from the cross-section of each cell was quantified (Figure 2A). From the red channel, both cells showed strong fluorescence at the exterior ends, indicating the cellular membrane while the interior had low fluorescence. When fluorescence intensity was determined at the green channel, the SYTO 9/FM 4–64 treated cells showed a bell-like intensity curve while the (KFF)_3_K-FAM/FM 4–64 treated cells showed a similar intensity curve with FM 4–64, only that the interior had a stronger fluorescence intensity (Figure 2A). To support our claims, we analyzed the (KFF)_3_K-FAM/TB and SYTO 9/TB treated cells and observed similar fluorescence intensity curves to the (KFF)_3_K-FAM/FM 4–64 and SYTO 9/FM 4–64 treated cells, respectively (Figure 2B). The (KFF)_3_K-FAM/TB and SYTO 9/TB treated cell images used as references were confirmed true by priorly capturing z-stack images using confocal microscopy (Supplementary Figure S2). Based on these results, we confirmed that (KFF)_3_K-FAM permeated into the cytoplasm of the cell. However, to our concern, (KFF)_3_K-FAM was also observed to localize or accumulate at the cellular membrane.

### Temperature Stimulates (KFF)_3_K-FAM Permeation

With (KFF)_3_K-FAM permeation efficiency only at approximately 50% and that localization of (KFF)_3_K-FAM observed at the cellular membrane, we tried to improve these conditions by looking at the abiotic factors that may have a direct effect to CPP internalization. Here we focused on temperature, incubation time, and (KFF)_3_K-FAM concentration. Starting with temperature, we found that (KFF)_3_K-FAM permeability was at its highest at temperatures ranging from 30 to 45°C (Supplementary Figure S3A). Setting the optimal temperature at 37°C, we subsequently optimized the incubation time. Although we began observing (KFF)_3_K-FAM permeation in *E. coli* at incubation times as low as 5 min, the percentage of permeated cells was also low. This gradually increased in conjunction to the increase in incubation time but began to maximize at incubation times longer than 60 min (Supplementary Figure S3B). Thus, setting the incubation time at 60 min, we performed the final optimization step, which was (KFF)_3_K-FAM concentration. For (KFF)_3_K-FAM concentration, we only observed a vast increase in (KFF)_3_K-FAM permeation from concentrations above 1.0 μM. Although (KFF)_3_K-FAM permeation gradually increased at concentrations higher than 1.0 μM, we noticed that too high concentrations of 4.0 μM and above resulted in higher accumulation of (KFF)_3_K-FAM at the cellular membrane and low signal-to-noise ratio (Supplementary Figure S3C).

**Figure 3:**
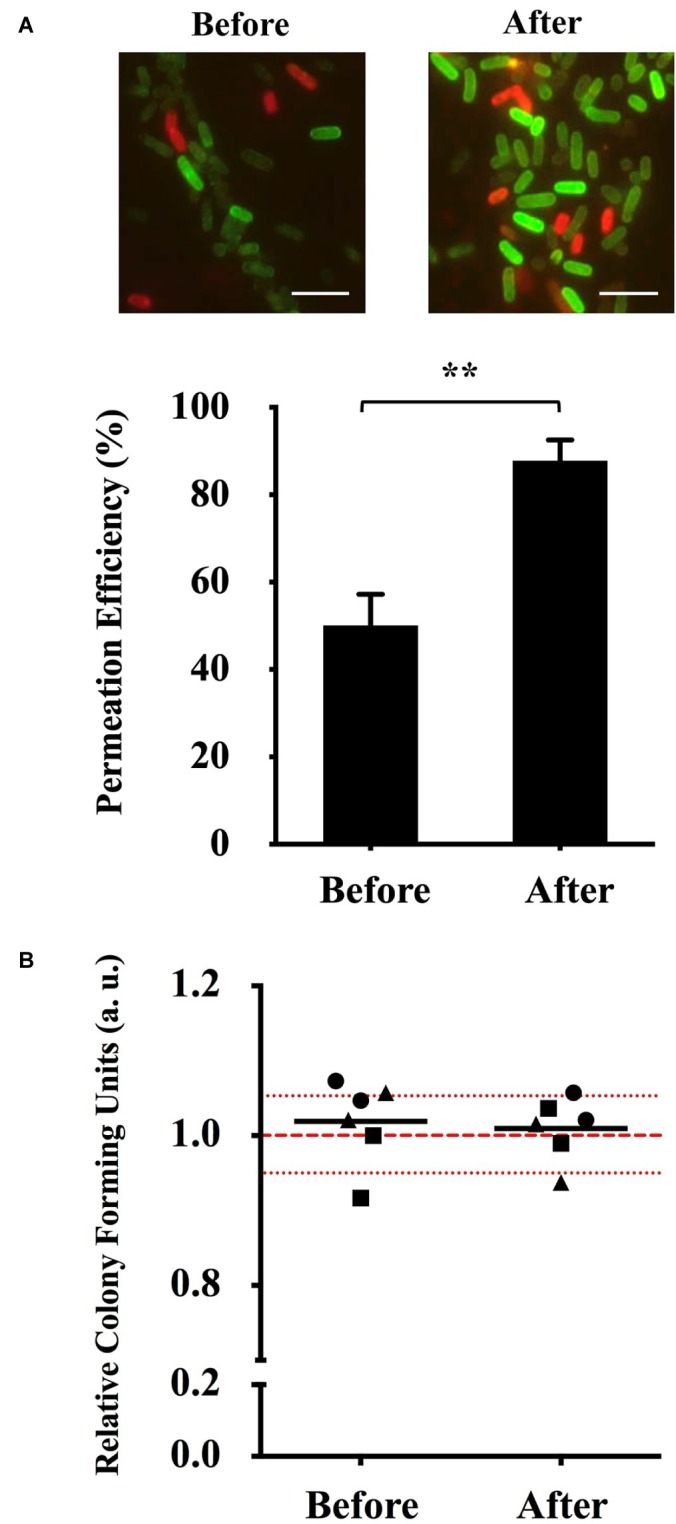
Optimization of (KFF)_3_K-FAM permeation and survival rate determination. **(A)** Fluorescence microscopic image (top) and permeation efficiency (bottom) of *E. coli* treated with (KFF)_3_K-FAM before (2 μM, RT, 1 h) and after (2 μM, 37°C, 1 h) optimization. Images of (KFF)_3_K-FAM permeated cells were captured at similar exposure times to allow for comparison. Scale bar is 5 μm. Statistical significance (*p* < 0.05) was determined by *t*-test with a *p* of 0.0016. **(B)** Relative colony forming units (CFUs) of (KFF)_3_K-FAM permeated cells sorted as single cells using flow cytometry before and after optimization. A total number of 240 cells/plate in doublets were sorted from three trials (● Trial 1, ■ Trial 2, ▲ Trial 3) and the relative average is indicated as the dark lines. All samples were treated with Trypan Blue prior to sorting. Non-(KFF)_3_K-FAM treated cells were sorted as the positive control and the average is represented as the red dotted line. The red spotted lines above and below the red dotted line represent the relative number of CFUs within the 5% range from the average.

To show the prominent increase of (KFF)_3_K-FAM permeation, we compared our new optimized conditions with those prior to optimization (Figure 3A). In addition to the clear difference between the fluorescence intensity of the cells observed with fluorescence microscopy, flow cytometry analysis also showed that cells treated with (KFF)_3_K-FAM at our new optimized conditions had a higher permeation efficiency of 85.1 ± 5.0% in comparison to cells that were treated at RT (48.1 ± 7.3%) (Supplementary Figure S1; after optimization). This 37% increase in efficiency suggests that temperature is an important factor for CPP internalization in *E. coli*. To further ensure that the increase in temperature does not have any direct effect on cell viability, single cells from each condition were sorted onto LB agarose plates. We showed that majority of the relative colony forming unit (CFU) values of sorted single cells over three trials were within the 5% range from the average of non-(KFF)_3_K-FAM treated cells (Figure 3B).

### (KFF)_3_K-FAM Permeability Can Be Promoted by Solution Tonicity

**Figure 4:**
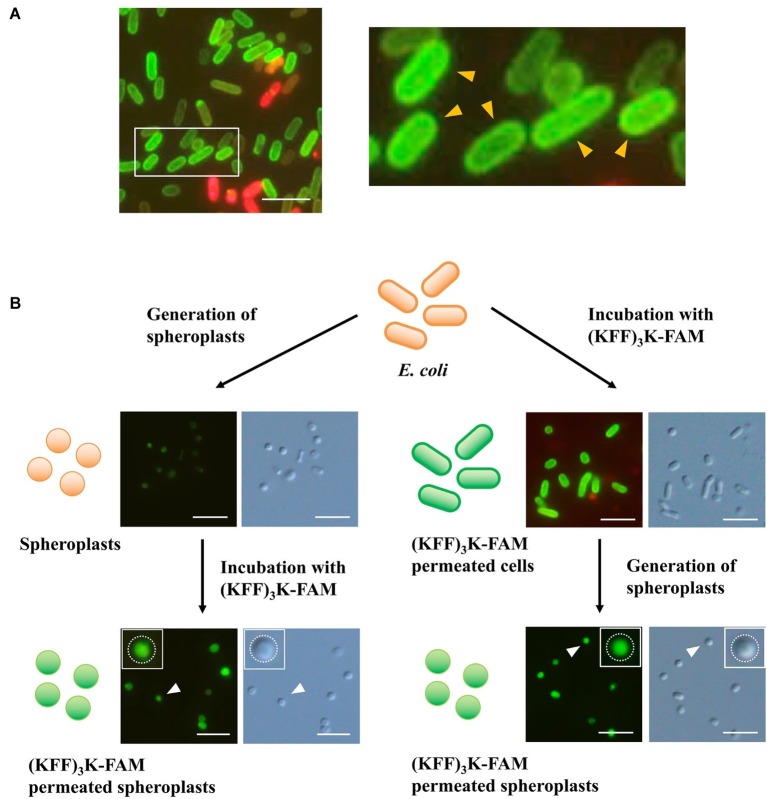
Evaluation (KFF)_3_K-FAM permeation in *E. coli via* spheroplast generation. **(A)** *E. coli* treated with 2 μM (KFF)_3_K-FAM at 37°C for 1 h and subsequently stained with 0.1% Trypan Blue. Inset, enlarged figure: *E. coli* permeated with (KFF)_3_K-FAM showing prominent accumulation at the lipid bilayer (yellow arrows). **(B)** Confirmation of (KFF)_3_K-FAM permeation before (right) and after (left) spheroplast generation. Successful generation of spheroplasts and (KFF)_3_K-FAM permeation was confirmed by bright field microscopy and fluorescence microscopy, respectively. Inset: images of single spheroplasts. White dotted lines represent the inner membrane bilayer. Scale bar is 5 μm.

Having proved that temperature was important to (KFF)_3_K-FAM permeation, we still faced the challenge of (KFF)_3_K-FAM accumulation at the cellular membrane (Figure 4A). Here, to ensure that we were looking at such a phenomenon, *E. coli* cells permeated with (KFF)_3_K-FAM were also tested with a higher TB concentration of 0.4% (Supplementary Figure S4). Our result shows that (KFF)_3_K-FAM permeated cells still showed membrane accumulation with higher TB concentrations validating our membrane accumulation observation. To identify the factor causing this accumulation, spheroplasts were used. Here, TB was excluded to show that no accumulation occurred at the inner lipid bilayer membrane. In the first approach, spheroplasts were generated and were subsequently treated with (KFF)_3_K-FAM. The circular shape of the *E. coli* cells suggests successful spheroplast generation (Figure 4B; left). Upon treatment with (KFF)_3_K-FAM, no accumulation of green fluorescence was observed at the inner membrane bilayer, but green fluorescence was observed intracellularly. In the second approach, the (KFF)_3_K-FAM treatment and spheroplasts formation steps were interchanged. As previously described, under conditions where the cellular membrane was intact, we observed the accumulation of (KFF)_3_K-FAM (Figure 4B; right). When spheroplasts were generated, in conjunction to the permeabilization of the outer membrane bilayer and degradation of the peptidoglycan layer, the membrane-accumulated (KFF)_3_K-FAM was no longer detectable. Only (KFF)_3_K-FAM that successfully permeated through the inner membrane bilayer was observed intracellularly. Based on this observation, we speculated that either the outer membrane bilayer or the peptidoglycan may be hindering efficient CPP permeation.

**Figure 5:**
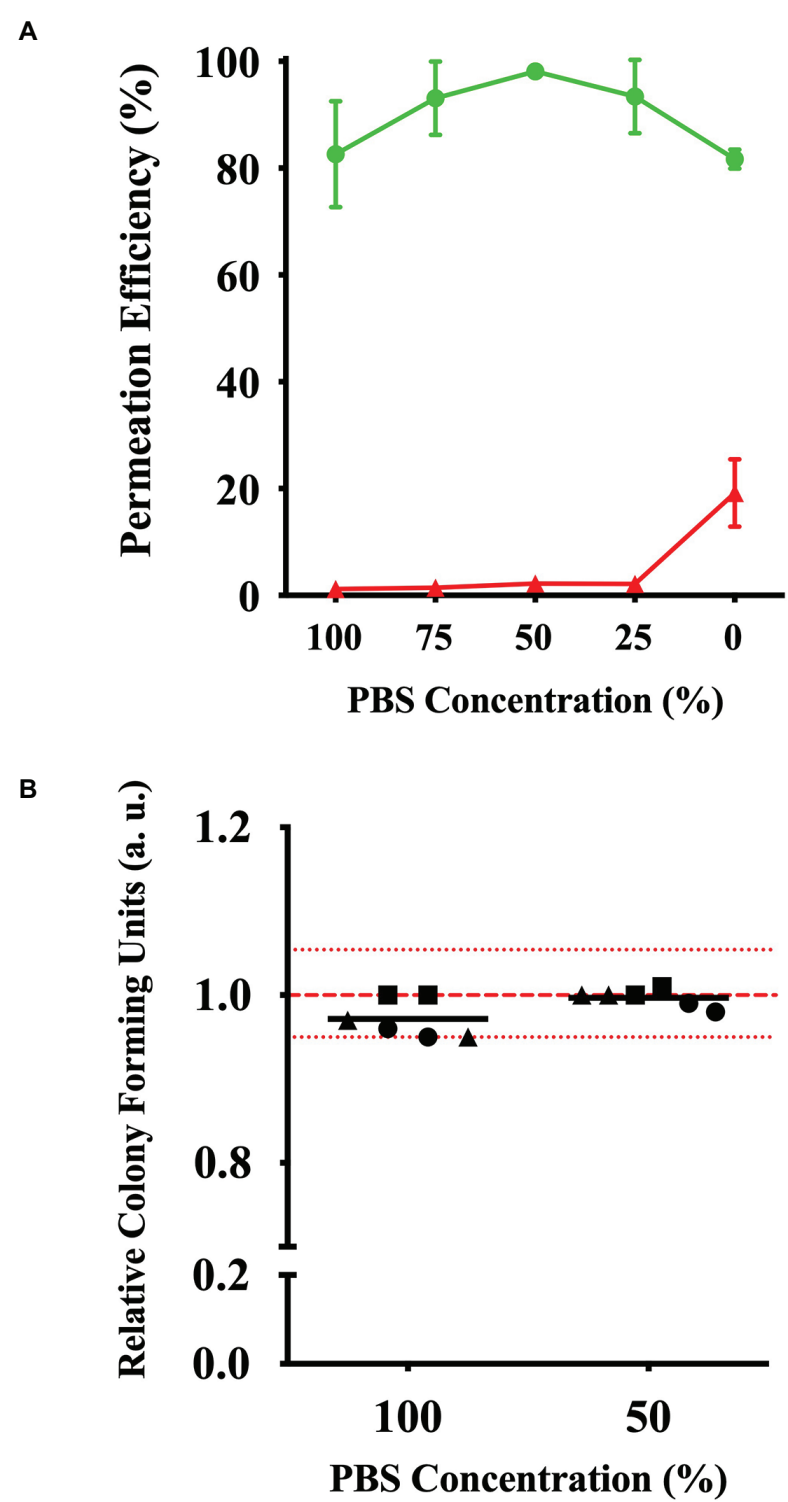
Effects of (KFF)_3_K-FAM permeability based on solution tonicity. **(A)** Permeation efficiency (green line) and dead cell population (red line) of *E. coli* treated with (KFF)_3_K-FAM incubated in 0–100% PBS concentrations. (KFF)_3_K-FAM incubation was performed with 2 μM concentrations at 37°C for 1 h. **(B)** Relative colony forming units (CFUs) of (KFF)_3_K-FAM permeated cells sorted as single cells using flow cytometry from 100 and 50% PBS concentration samples. A total number of 240 cells/plate in doublets were sorted and the relative average is indicated as the dark lines. All samples were treated with Trypan Blue prior to sorting. Non-(KFF)_3_K-FAM treated cells were sorted as a positive control and the average is represented as the red dotted line. The red spotted lines above and below the red dotted line represent the relative number of CFUs within the 5% range from the average.

With the possibility of the peptidoglycan layer limiting efficient CPP permeation into the cytoplasm, we evaluated the effects of solution tonicity during incubation. Based on the incubation of the *E. coli* cells with (KFF)_3_K-FAM in 0–100% PBS, flow cytometry showed that (KFF)_3_K-FAM permeability gradually increased from 82.2 ± 10.5% as the concentration of PBS decreased and peaked at the highest efficiency at 98.1 ± 1.1% when dilution was at 50% (Figure 5A). As PBS was further diluted, we observed that the permeation efficiency began to decrease resulting in a 78.2 ± 5.8% efficiency in H_2_O (0% PBS) only. Here, the dead cell population was at 19.2 ± 6.3%. At 50% PBS, the dead cell population was only at 2.2 ± 1.5%. Interestingly, in addition to highly efficient permeability, we also observed that the fluorescence of (KFF)_3_K-FAM permeated cells was more uniform at 50% PBS due to the narrow and sharp peak from our flow cytometric analysis (Supplementary Figure S5). Based on these results, we concluded that solution tonicity does have an effect to CPP permeation. Simultaneously, to ensure that cell *via*bility is not compromised by osmotic stress, single cells incubated with (KFF)_3_K-FAM in 50 and 100% PBS were sorted. The relative CFU values for three trials observed within the 5% range from the average of the live non-(KFF)_3_K-FAM treated cells showed commendable cell viability (Figure 5B).

### Optimized Abiotic Conditions Improved (KFF)_3_K-FAM Permeation in Various Bacterial Strains

**Figure 6:**
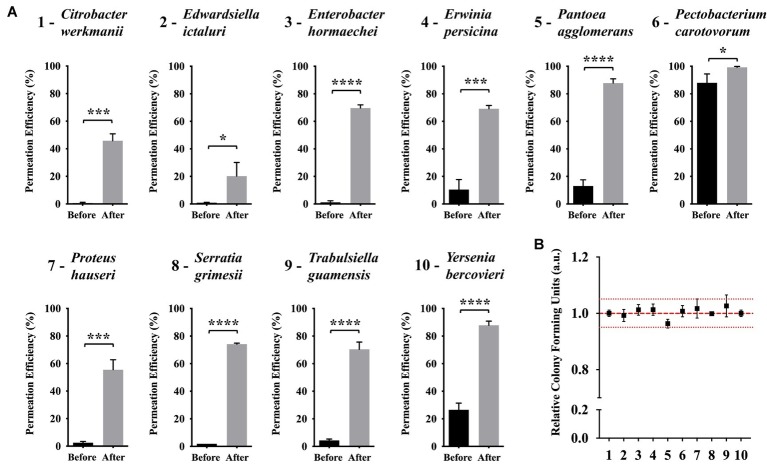
Efficiency of (KFF)_3_K-FAM permeation in various bacterial strains under conditions before and after optimization. **(A)** Bacterial strains tested were taxonomically close to *E. coli* from the Enterobacteriaceae family. Statistical significance (*p* < 0.05) was determined by *t*-test where * indicates the level of significance. (KFF)_3_K-FAM permeation was performed with conditions before (0.4 μM, 23°C, 1 h, 100% PBS) and after (0.4 μM, OT, 1 h, 50% PBS, OT, optimal growth temperature) optimization followed by 0.1% Trypan Blue staining **(B)** Relative colony forming units (CFUs) of (KFF)_3_K-FAM permeated cells sorted as single cells using flow cytometry after optimization. The numbers at the X-axis correspond to the bacterial strains presented in a. A total number of 240 cells/plates were sorted from three trials and the relative average is presented. Non-(KFF)_3_K-FAM treated cells were sorted as a positive control and the average is represented as the red dotted line. The red spotted lines above and below the red dotted line represent the relative number of CFUs within the 5% range from the average.

Based on our results on the effects and optimization of the abiotic factors to improve (KFF)_3_K-FAM permeation efficiency, we showed that temperature and solution tonicity were important factors for *E. coli*. Since our goal is to investigate the possibility of employing CPP with diverse bacterial strains, we subsequently decided to test if (KFF)_3_K-FAM was applicable with other bacterial strains and whether these abiotic factors could also improve its permeation. To do this, we compared the permeation of (KFF)_3_K-FAM with bacteria from the Enterobacteriaceae family based on conditions before and after optimization *via* flow cytometric analysis. Here, the concentration of (KFF)_3_K-FAM used during incubation was 0.4 μM. Also note that, with the exception of *Erwinia, Pantoea,* and *Pectobacterium* (Patel et al., 2017), seven of the bacteria tested come from genera that have not been treated with CPP. Overall, we observed that (KFF)_3_K-FAM could permeate into all the bacterial strains at conditions before optimization but the efficiency was very low (Figure 6A). With the exception of *P. carotovorum*, which showed a high permeation efficiency of 88.0 ± 6.5%, all the other strains had efficiencies below 30%. In fact, six out of the 10 strains showed efficiencies below 5%. However, this was a drastic difference when our optimized conditions were employed. In all the strains, we saw a huge increment in (KFF)_3_K-FAM permeation where the five strains *E. hormaechei*, *P. agglomerans*, *S. grimesii*, *T. guamensis*, and *Y. bercovieri* showed the most prominent increase ranging between 60 and 75%. This was followed by *C. werkmanii*, *E. persicina*, and *P. hauseri* between 50 and 60% and *E. ictaluri* and *P. carotovorum* below 20%. Fluorescence microscopy was also used to confirm (KFF)_3_K-FAM internalization (Supplementary Figure S6). Furthermore, to ensure that this increment did not have any cytotoxic effect on the host, single cells from each bacterial strain were sorted and the relative CFUs were counted. As a result, the number of CFUs attained for all bacterial strains were equivalent to that of non-(KFF)_3_K-FAM treated controls. Based on this result, we concluded that in addition to *E. coli*, the optimization of abiotic factors such as temperature and solution tonicity can greatly promote the permeation and application of CPP with other bacterial strains.

## Discussion

Differing from the current major application of CPPs as carriers to cargo ASOs where its efficiency is driven by strain compatibility, our delivery system for the development of a highly efficient mutagenesis protocol for novel and hard-to-transform strains is directed toward strain diversity. Thus, a delivery system that would be applicable to all bacterial strains would be required. Although CPPs have the ability to penetrate through the bacterial membrane, we have learned that the permeability of CPPs is known to differ among various bacterial strains. A valid explanation to this difference could be due to the complex structure of the bacterial cellular membrane. Not only differing in the number of the lipid-bilayer membranes and thickness of the peptidoglycan layer, gram-negative and gram-positive strains also harbor unique components such as lipopolysaccharides (LPSs) and techoic acids respectively. Furthermore, to complicate matters, it is also known that different bacterial species harbor different membrane compositions (Sohlenkamp and Geiger, 2016).

Intensive research on CPP permeation in mammalian systems has suggested that factors such as CPP biophysical property (Lindgren and Langel, 2011; Mickan et al., 2014), CPP-cargo properties (Zorko and Langel, 2005), and CPP-cellular membrane interactions (Eiriksdottir et al., 2010) strongly contribute to the efficacy of CPP permeability, but most of these researches are based on the internalization of CPP through a single lipid-bilayer membrane. Efforts to understand CPP permeation in bacteria have been performed but thus far are only limited to several reports. For example, one of the most detailed studies was performed using the (KFF)_3_K peptide conjugated to peptide nucleic acids (PNAs) where biomarkers and indicators were used to evaluate the penetration of the (KFF)_3_K-PNA conjugates through the LPS layer and the outer and inner lipid bilayer of *E. coli* (Eriksson et al., 2002). Other attempts to perform comparative studies on the permeation of CPPs with specific strains (Abushahba et al., 2016; Xue et al., 2018) or the identification of specific peptide sequence motifs for efficient cargo delivery by screening large CPP libraries (Oikawa et al., 2018) have also been recently reported. As such, although we are beginning to understand CPP permeation in bacteria, the findings in these researches are limited to specific bacterial strains and the conditions may not be applicable to the development of our delivery system.

Therefore, to establish a delivery system applicable to diverse bacterial strains, we needed to identify common factors shared among bacteria that may promote CPP internalization. Using *E. coli* and the (KFF)_3_K CPP as models, we first looked into the three major factors that may have a direct impact. Among these factors, we were pleased to find that temperature showed a prominent effect by the marked increase of permeability efficiency by approximately 40% (Figure 3). In mammalian cells, temperature has been noted as a factor that effects CPP permeation where higher efficiency was observed at higher temperatures. This is known to be related to the increase in cellular metabolism resulting in the engulfment of CPP *via* endocytosis (Richard et al., 2003). Since most bacteria do not perform endocytosis, we speculated that CPP permeation had to be a direct interaction between CPP and the cellular membrane. It has been reported that the state of the lipid bilayer membrane is closely related to changes in temperature where at lower temperatures, membranes are more rigid while at higher temperatures, they are considered more fluidic (Los and Murata, 2004). Based on these studies and in reference to our results (Figure 3, Supplementary Figure S3), we therefore speculate that higher temperature results in a less rigid membrane, thus allowing CPP to permeate better through the cellular membrane.

In the process of optimizing the necessary conditions to promote CPP permeation, we were intrigued to see that accumulation of (KFF)_3_K-FAM occurred at the cellular membrane (Figures 2, 4A, Supplementary Figures S3, S4). Assuming that if membrane fluidity is a major factor promoting CPP permeability at elevated temperatures, accumulation of (KFF)_3_K-FAM should not be prominent. Furthermore, since we already know that (KFF)_3_K can permeate through the outer LPS layer and the inner and outer lipid bilayer membranes of *E. coli* (Eriksson et al., 2002), one possible explanation to this phenomenon may be the peptidoglycan layer. The peptidoglycan layer, being a rigid and mesh-like layer found in all bacteria comprised of glycan-strands cross-linked by short peptides that provides structural strength and shape (Vollmer et al., 2008), could be limiting the rate of CPP permeation, hence the accumulation of (KFF)_3_K-FAM. Another explanation could be the affinity of FAM with the lipid bilayer membrane (Hedegaard et al., 2018) but we think that the effects would be small since we did not see FAM accumulation when *E. coli* spheroplasts were treated with (KFF)_3_K-FAM (Figure 4B; left). Therefore, we tried to experimentally validate the former speculation by first using spheroplasts. Since spheroplasts are generated by permeabilizing the outer membrane and using lysozyme to degrade the peptidoglycan layer, we interchanged the (KFF)_3_K-FAM permeation steps before and after spheroplast generation to observe (KFF)_3_K-FAM accumulation. Interestingly, we observed that the accumulation of (KFF)_3_K-FAM was exclusive to the cells treated with the conjugate before spheroplasts generation (Figure 4). Showing that the peptidoglycan layer may be the cause of (KFF)_3_K-FAM accumulation, we next tested this observation by evaluating CPP permeation under different solution tonicity conditions. Changing solution tonicity conditions causes the peptidoglycan layer to expand allowing larger molecules to diffuse through the gaps of the peptidoglycan molecules, thus, resulting in more efficient CPP permeation. We were pleased to observe that higher solution tonicity resulted in higher (KFF)_3_K-FAM permeation efficiency, stronger fluorescence, and better permeation uniformity among the cells (Figure 5, Supplementary Figure S5). Although we were unable to show the direct molecular interaction between the peptidoglycan layer and (KFF)_3_K-FAM, from our results, we could say that there could be a strong relation between this layer and CPP permeation. Further experimentation such as altering the CPP sequences and properties or evaluating the peptidoglycan composition using different bacterial strains could further enlighten us on this possible interaction.

Finally, to test if our optimized conditions based on temperature and solution tonicity does improve or allow efficient CPP permeation in diverse bacteria, bacteria from the Enterobacteriaceae family were tested. Bacterial strains from this family were chosen as model strains primarily because they were phylogenetically related to *E. coli*, but it was also because many strains classified in this family were either members of the gut microbiota or important disease-causing pathogens showing medical relevance. As shown, our conditions were effective against all 10 strains with improved permeation efficiency of above 20% (Figure 6). Furthermore, with seven out of the 10 strains coming from genera that are newly tested with CPP, we show that our conditions also promote CPP internalization in non-CPP tested strains. We believe that this evaluation is a huge progress in our goal to establish a delivery system targeting novel or hard-to-transform strains. However, it is important to take note that although we showed that a single CPP can be employed with numerous bacterial strains within a given family, it does not confirm that with our conditions, (KFF)_3_K-FAM would show high permeability with bacteria from different phyla. Assuming that cellular membrane composition does play a major role in CPP permeation, the use of more compatible CPPs would be required. In addition, we have only provided examples of gram-negative bacterial strains but have not evaluated CPP permeation under our optimized conditions with gram-positive strains. We believe this would be a challenge since gram-positive strains have a thicker peptidoglycan layer with possible different composition to its gram-negative counterparts.

In summary, we showed that evaluation and optimization of abiotic factors such as temperature and solution tonicity promote CPP permeation. Furthermore, such factors are easy to optimize and theoretically can be applied to all bacterial strains without influencing cell viability. Our results also suggest that CPP internalization is not solely dependent on the biophysical properties of CPP but can be enhanced by other factors allowing for a single CPP to be applicable with diverse bacterial strains. Hence, our data suggest the flexibility and potential of CPPs as an efficient system for the delivery of biomolecules into novel or hard-to-transform strains. We hope to subsequently challenge the permeation of (KFF)_3_K-FAM with other bacterial strains from different phyla including gram-positive strains and proceed with the establishment of characterized or functional mutants using CPP by addressing the remaining unresolved major issues.

## Data Availability Statement

All datasets generated for this study are included in the article/Supplementary Material.

## Author Contributions

DT performed most of the experiments and analyses. YY and GI supported some of the experimental work. TMu and TMo designed the study. TMo wrote the manuscript.

### Conflict of Interest

The authors declare that the research was conducted in the absence of any commercial or financial relationships that could be construed as a potential conflict of interest.

## References

[ref1] Abushahba M. F. Mohammad H. Thangamani S. Hussein A. A. Seleem M. N. (2016). Impact of different cell penetrating peptides on the efficacy of antisense therapeutics for targeting intracellular pathogens. Sci. Rep. 6:20832. 10.1038/srep20832, PMID: 26860980 PMC4748415

[ref2] Aune T. E. Aachmann F. L. (2010). Methodologies to increase the transformation efficiencies and the range of bacteria that can be transformed. Appl. Microbiol. Biotechnol. 85, 1301–1313. 10.1007/s00253-009-2349-1, PMID: 19946685

[ref3] Avci F. G. Akbulut B. S. Ozkirimli E. (2018). Membrane active peptides and their biophysical characterization. Biomol. Ther. 8, pii: E77. 10.3390/biom8030077, PMID: 30135402 PMC6164437

[ref4] Benincasa M. Pacor S. Gennaro R. Scocchi M. (2009). Rapid and reliable detection of antimicrobial peptide penetration into gram-negative bacteria based on fluorescence quenching. Antimicrob. Agents Chemother. 53, 3501–3504. 10.1128/AAC.01620-08, PMID: 19470515 PMC2715650

[ref5] Borrelli A. Tornesello A. L. Tornesello M. L. Buonaguro F. M. (2018). Cell penetrating peptides as molecular carriers for anti-cancer agents. Molecules 23, pii: E295. 10.3390/molecules23020295, PMID: 29385037 PMC6017757

[ref6] Cabezon E. Ripoll-Rozada J. Pena A. De La Cruz F. Arechaga I. (2015). Towards an integrated model of bacterial conjugation. FEMS Microbiol. Rev. 39, 81–95. 10.1111/1574-6976.12085, PMID: 25154632

[ref7] Deshmukh K. Ramanan S. R. Kowshik M. (2019). Novel one step transformation method for *Escherichia coli* and *Staphylococcus aureus* using arginine-glucose functionalized hydroxyapatite nanoparticles. Mater. Sci. Eng. C Mater. Biol. Appl. 96, 58–65. 10.1016/j.msec.2018.10.088, PMID: 30606568

[ref8] Eiriksdottir E. Konate K. Langel U. Divita G. Deshayes S. (2010). Secondary structure of cell-penetrating peptides controls membrane interaction and insertion. Biochim. Biophys. Acta 1798, 1119–1128. 10.1016/j.bbamem.2010.03.005 20214875

[ref9] Eriksson M. Nielsen P. E. Good L. (2002). Cell permeabilization and uptake of antisense peptide-peptide nucleic acid (PNA) into *Escherichia coli*. J. Biol. Chem. 277, 7144–7147. 10.1074/jbc.M106624200, PMID: 11739379

[ref10] Giedyk M. Jackowska A. Rownicki M. Kolanowska M. Trylska J. Gryko D. (2019). Vitamin B12 transports modified RNA into *E. coli* and *S. typhimurium* cells. Chem. Commun. 55, 763–766. 10.1039/C8CC05064C, PMID: 30480264

[ref11] Goessweiner-Mohr N. Arends K. Keller W. Grohmann E. (2014). Conjugation in gram-positive bacteria. Microbiol. Spectr. 2:PLAS-0004-2013. 10.1128/microbiolspec.plas-0004-2013 26104193

[ref12] Gomarasca M. Martins T. F. C. Greune L. Hardwidge P. R. Schmidt M. A. Ruter C. (2017). Bacterium-derived cell-penetrating peptides deliver gentamicin to kill intracellular pathogens. Antimicrob. Agents Chemother. 61, pii: e02545-16. 10.1128/AAC.02545-16, PMID: 28096156 PMC5365713

[ref13] Gonzalez-Paredes A. Sitia L. Ruyra A. Morris C. J. Wheeler G. N. Mcarthur M.. (2019). Solid lipid nanoparticles for the delivery of anti-microbial oligonucleotides. Eur. J. Pharm. Biopharm. 134, 166–177. 10.1016/j.ejpb.2018.11.017, PMID: 30468838

[ref14] Good L. Awasthi S. K. Dryselius R. Larsson O. Nielsen P. E. (2001). Bactericidal antisense effects of peptide-PNA conjugates. Nat. Biotechnol. 19, 360–364. 10.1038/86753, PMID: 11283595

[ref15] Guidotti G. Brambilla L. Rossi D. (2017). Cell-penetrating peptides: from basic research to clinics. Trends Pharmacol. Sci. 38, 406–424. 10.1016/j.tips.2017.01.003, PMID: 28209404

[ref16] Harrisson F. Callebaut M. Vakaet L. (1981). Microspectrographic analysis of trypan blue-induced fluorescence in oocytes of the Japanese quail. Histochemistry 72, 563–578. 10.1007/BF00493276, PMID: 7298390

[ref17] Hedegaard S. F. Derbas M. S. Lind T. K. Kasimova M. R. Christensen M. V. Michaelsen M. H.. (2018). Fluorophore labeling of a cell-penetrating peptide significantly alters the mode and degree of biomembrane interaction. Sci. Rep. 8:6327. 10.1038/s41598-018-24154-z, PMID: 29679078 PMC5910404

[ref18] Kotnik T. Frey W. Sack M. Haberl Meglic S. Peterka M. Miklavcic D. (2015). Electroporation-based applications in biotechnology. Trends Biotechnol. 33, 480–488. 10.1016/j.tibtech.2015.06.002, PMID: 26116227

[ref19] Kristensen M. Birch D. Morck Nielsen H. (2016). Applications and challenges for use of cell-penetrating peptides as delivery vectors for peptide and protein cargos. Int. J. Mol. Sci. 17, pii: E185. 10.3390/ijms17020185, PMID: 26840305 PMC4783919

[ref20] Lindgren M. Hallbrink M. Prochiantz A. Langel U. (2000). Cell-penetrating peptides. Trends Pharmacol. Sci. 21, 99–103. 10.1016/S0165-6147(00)01447-4, PMID: 10689363

[ref21] Lindgren M. Langel U. (2011). Classes and prediction of cell-penetrating peptides. Methods Mol. Biol. 683, 3–19. 10.1007/978-1-60761-919-2_1 21053118

[ref22] Liu H. Price M. N. Waters R. J. Ray J. Carlson H. K. Lamson J. S.. (2018). Magic pools: parallel assessment of transposon delivery vectors in bacteria. mSystems 3, pii: e00143-17. 10.1128/mSystems.00143-17, PMID: 29359196 PMC5768790

[ref23] Los D. A. Murata N. (2004). Membrane fluidity and its roles in the perception of environmental signals. Biochim. Biophys. Acta 1666, 142–157. 10.1016/j.bbamem.2004.08.002 15519313

[ref24] Mickan A. Sarko D. Haberkorn U. Mier W. (2014). Rational design of CPP-based drug delivery systems: considerations from pharmacokinetics. Curr. Pharm. Biotechnol. 15, 200–209. 10.2174/138920101503140822101814, PMID: 25312539

[ref25] Monk I. R. Shah I. M. Xu M. Tan M. W. Foster T. J. (2012). Transforming the untransformable: application of direct transformation to manipulate genetically *Staphylococcus aureus* and *Staphylococcus epidermidis*. MBio 3, pii: e00277-11. 10.1128/mBio.00277-11, PMID: 22434850 PMC3312211

[ref26] Oikawa K. Islam M. M. Horii Y. Yoshizumi T. Numata K. (2018). Screening of a cell-penetrating peptide library in *Escherichia coli*: relationship between cell penetration efficiency and cytotoxicity. ACS Omega 3, 16489–16499. 10.1021/acsomega.8b02348

[ref27] Patel R. R. Sundin G. W. Yang C. H. Wang J. Huntley R. B. Yuan X.. (2017). Exploration of using antisense peptide nucleic acid (PNA)-cell penetrating peptide (CPP) as a novel bactericide against fire blight pathogen *Erwinia amylovora*. Front. Microbiol. 8:687. 10.3389/fmicb.2017.00687, PMID: 28469617 PMC5395615

[ref28] Rajarao G. K. Nekhotiaeva N. Good L. (2002). Peptide-mediated delivery of green fluorescent protein into yeasts and bacteria. FEMS Microbiol. Lett. 215, 267–272. 10.1111/j.1574-6968.2002.tb11401.x, PMID: 12399045

[ref29] Ramsey J. D. Flynn N. H. (2015). Cell-penetrating peptides transport therapeutics into cells. Pharmacol. Ther. 154, 78–86. 10.1016/j.pharmthera.2015.07.003, PMID: 26210404

[ref30] Richard J. P. Melikov K. Vives E. Ramos C. Verbeure B. Gait M. J.. (2003). Cell-penetrating peptides. A reevaluation of the mechanism of cellular uptake. J. Biol. Chem. 278, 585–590. 10.1074/jbc.M209548200, PMID: 12411431

[ref31] Sohlenkamp C. Geiger O. (2016). Bacterial membrane lipids: diversity in structures and pathways. FEMS Microbiol. Rev. 40, 133–159. 10.1093/femsre/fuv008, PMID: 25862689

[ref32] Sun Y. Sun T. L. Huang H. W. (2014). Physical properties of *Escherichia coli* spheroplast membranes. Biophys. J. 107, 2082–2090. 10.1016/j.bpj.2014.09.034, PMID: 25418093 PMC4223228

[ref33] Vollmer W. Blanot D. De Pedro M. A. (2008). Peptidoglycan structure and architecture. FEMS Microbiol. Rev. 32, 149–167. 10.1111/j.1574-6976.2007.00094.x, PMID: 18194336

[ref34] Wang P. Yu Z. Li B. Cai X. Zeng Z. Chen X.. (2015). Development of an efficient conjugation-based genetic manipulation system for *Pseudoalteromonas*. Microb. Cell Factories 14:11. 10.1186/s12934-015-0194-8, PMID: 25612661 PMC4318363

[ref35] Xue X. Y. Mao X. G. Zhou Y. Chen Z. Hu Y. Hou Z.. (2018). Advances in the delivery of antisense oligonucleotides for combating bacterial infectious diseases. Nanomedicine 14, 745–758. 10.1016/j.nano.2017.12.026, PMID: 29341934

[ref36] Yosef I. Goren M. G. Globus R. Molshanski-Mor S. Qimron U. (2017). Extending the host range of bacteriophage particles for DNA transduction. Mol. Cell 66, 721–728.e723. 10.1016/j.molcel.2017.04.025 28552617

[ref37] Zorko M. Langel U. (2005). Cell-penetrating peptides: mechanism and kinetics of cargo delivery. Adv. Drug Deliv. Rev. 57, 529–545. 10.1016/j.addr.2004.10.010, PMID: 15722162

