# Supplementary material for: Abiotic Factors Promote Cell Penetrating Peptide Permeability in Enterobacteriaceae Models
Source: Front Microbiol. 2019 Nov 26;10:2534. doi: 10.3389/fmicb.2019.02534 (PMC6902036; doi:10.3389/fmicb.2019.02534)
Supplement: Supplementary file 1 [file Image_1.pdf]

Supplementary Material

Abiotic factors promote cell penetrating peptide permeability in Enterobacteriaceae models

Daichi Toyohara[1], Yasuhito Yokoi[1], Go Inoue[2], Takahiro Muraoka[2], Tetsushi Mori[1]*

[1] Department of Biotechnology and Life Science, Tokyo University of Agriculture and Technology, 2-24-16 Naka-cho, Koganei, Tokyo 184-8588, Japan.

[2] Department of Organic and Polymer Materials Chemistry, Tokyo University of Agriculture and Technology, 2-24-16 Naka-cho, Koganei, Tokyo 184-8588, Japan.

*** Correspondence:**

Tetsushi Mori

moritets@go.tuat.ac.jp

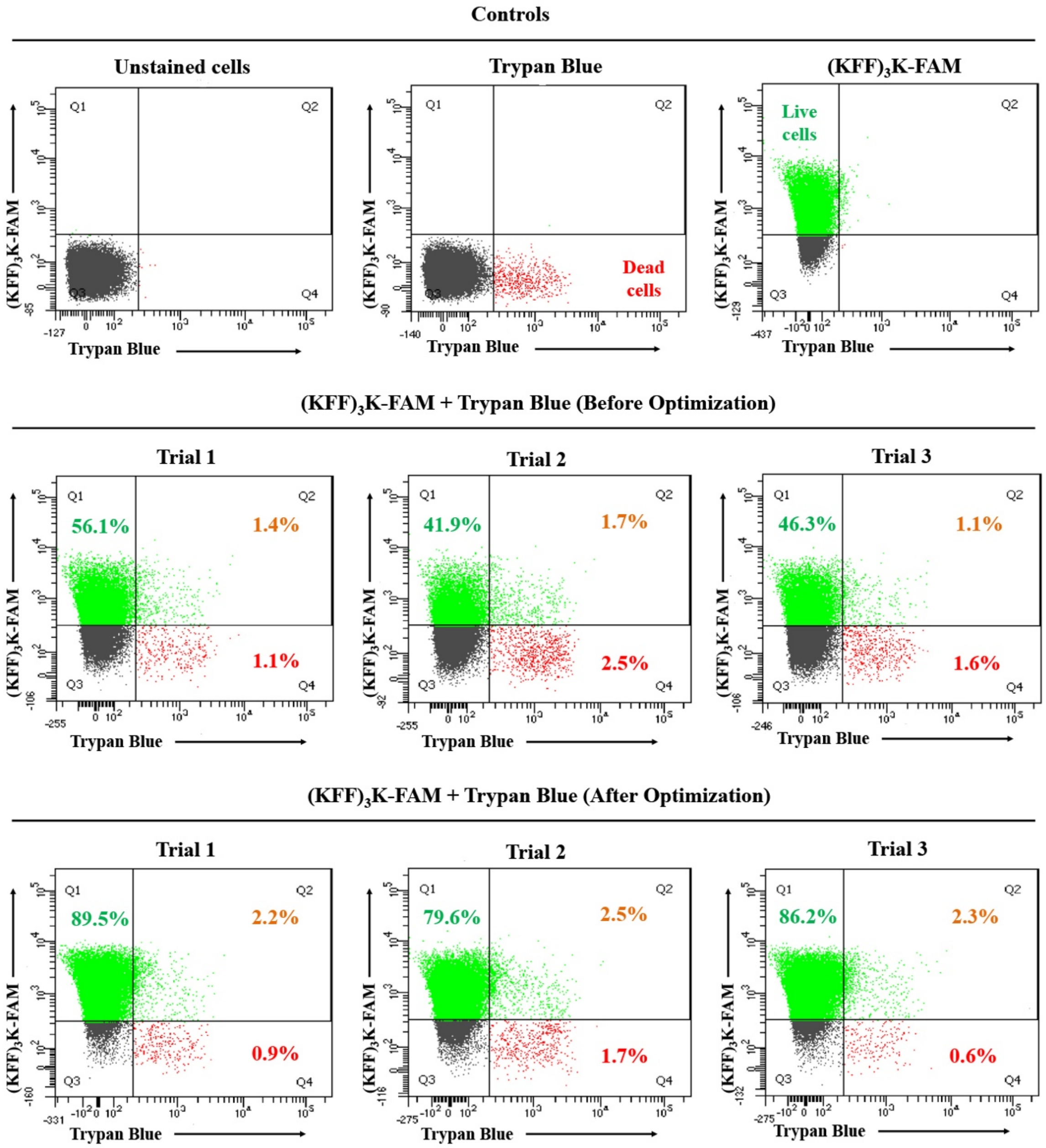

Fig. S1 Flow cytometric analysis of $(KFF)_3K$-FAM permeated *E. coli* before and after permeation assay condition optimization. $(KFF)_3K$-FAM permeation was performed at 2 μM concentration in PBS at room temperature (23°C; Before) and 37°C (After) for 1 h and subsequently stained with 0.1% Trypan Blue (TB). Untreated cells, cells stained only with TB and cells treated only with $(KFF)_3K$-FAM were used as controls. Fluorescence compensation was performed using the auto compensation feature of the BD FACSDiva™ software provided with the cell sorter system.

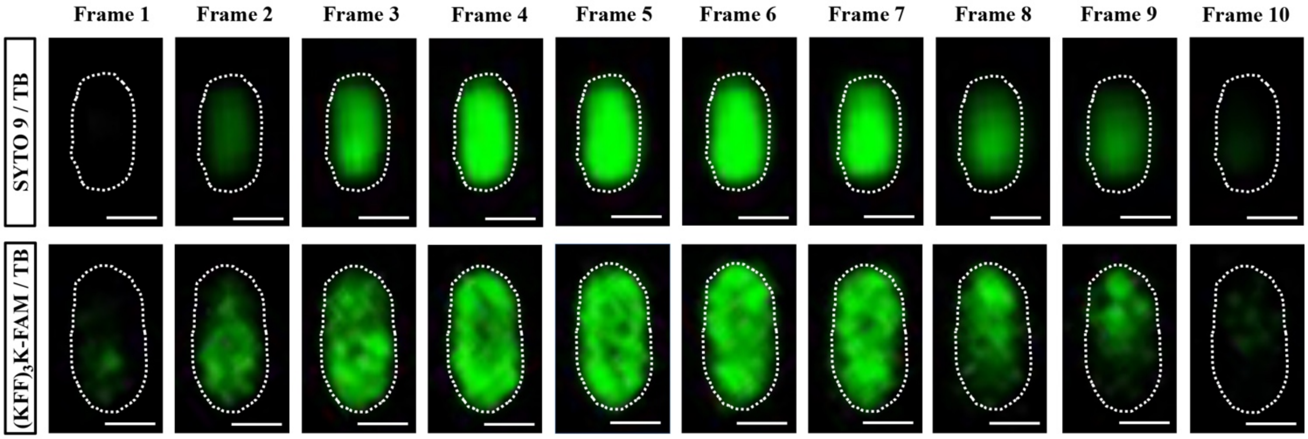

Fig. S2 Z-stack fluorescence microscopic images of *E. coli* cells permeated with SYTO 9 and $(KFF)_3$K-FAM, respectively. $(KFF)_3$K-FAM permeation was performed at 2 µM concentration in PBS at room temperature while SYTO 9 staining was performed based on the manufacturer's protocol. Both samples were stained with 0.1% Trypan Blue (TB) prior to microscopic observation. Z-stack images were captured with the Fluoview FV1000 confocal microscope (Olympus Corporation, Tokyo, Japan) equipped with a 100X objective lens and an Alexa Fluor 488 filter for FAM fluorescence detection. Each frame (1-10) was captured at 0.2 µm intervals to validate the localization of $(KFF)_3$K-FAM and SYTO 9. At Frames 5 and 6 (approximately 1.0 µm cross-section of the cells), we observed that $(KFF)_3$K-FAM, in addition to permeation into the cytoplasm, also accumulated at the cellular membrane (outlined as white dotted lines). SYTO 9 on the other hand showed complete permeation into the cytoplasm. Scale bar is 2 µm.

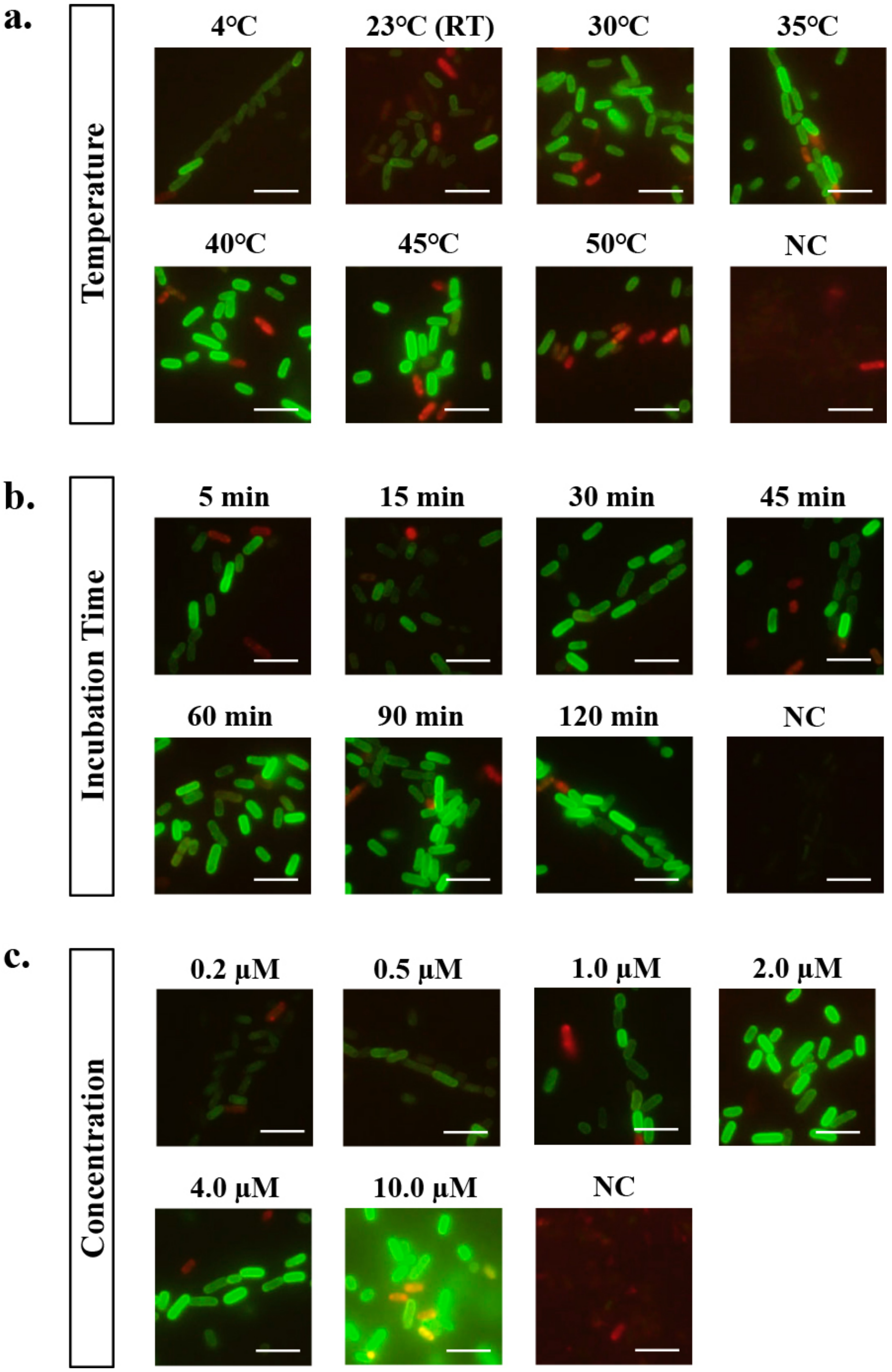

Fig. S3 Optimization of $(KFF)_3K$-FAM permeation into *E. coli* based on 3 abiotic factors, a. temperature, b. incubation time and c. $(KFF)_3K$-FAM concentration. Optimization was performed sequentially and the optimal condition for each optimized factor was employed in the subsequent optimization step. $(KFF)_3K$-FAM untreated cells stained with Trypan Blue at each condition was used as negative controls (NC). Scale bar is 5 µm.

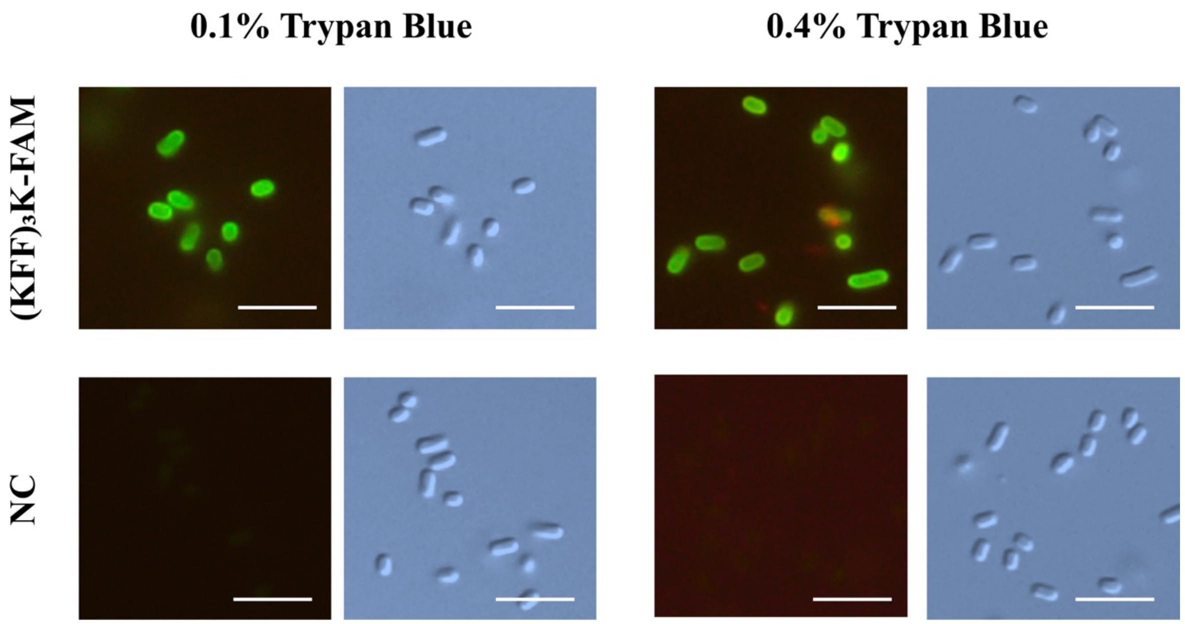

Fig. S4 Fluorescence and bright field microscopic images of $(KFF)_3K$-FAM permeated *E. coli* cells treated with 0.1% and 0.4% Trypan Blue (TB) concentration. $(KFF)_3K$-FAM permeation was performed based on the conditions after optimization (2 µM, OT, 1 h, 50% PBS; OT: optimal growth temperature). $(KFF)_3K$-FAM untreated cells stained with TB at each condition was used as negative controls (NC). Images of $(KFF)_3K$-FAM permeated cells and the negative controls were captured at similar exposure times to allow for comparison. Scale bar is 5 µm.

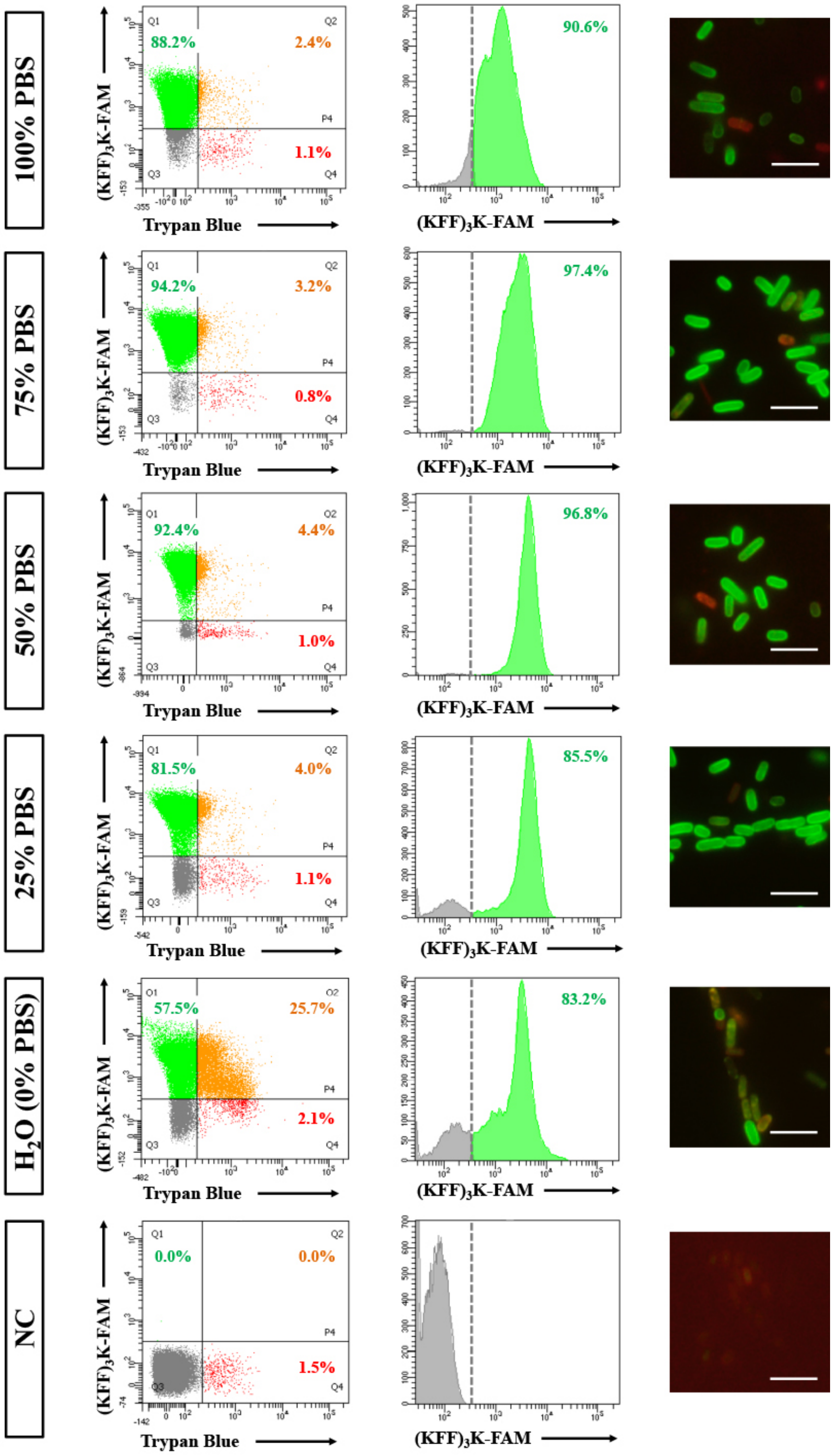

Fig. S5 Flow cytometric analysis of (KFF)$_3$K-FAM permeated cells at 0 (H_2O) – 100% PBS. (KFF)$_3$K-FAM incubation was performed with 2 μM concentrations at 37°C for 1 h followed by 0.1% Trypan Blue (TB) staining. Non-(KFF)$_3$K-FAM permeated cells were used as the negative control (NC). (KFF)$_3$K-FAM permeation was further confirmed by fluorescence microscopy. Scale bar is 5 μm.

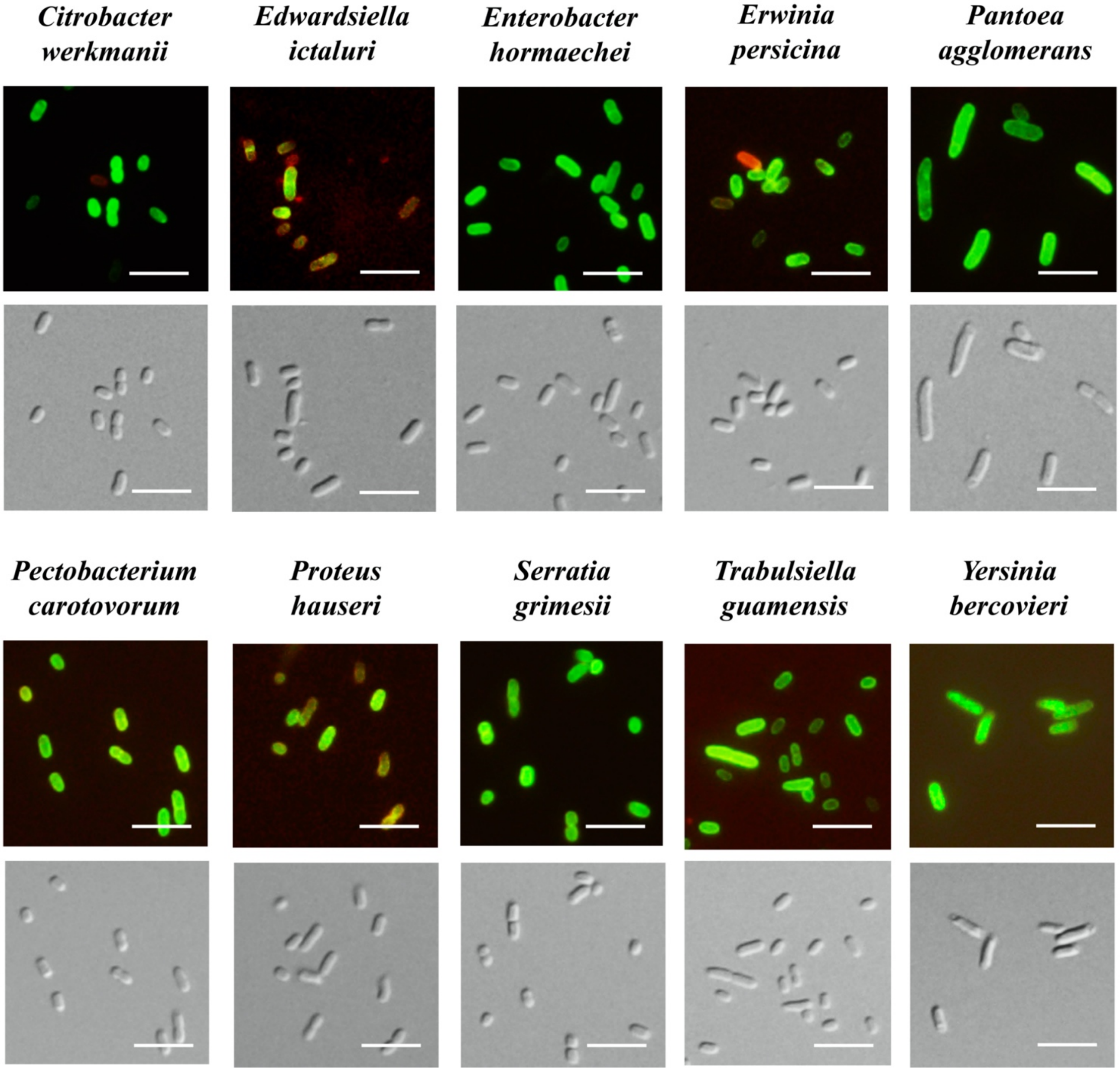

Fig. S6 Fluorescence and bright field microscopic images of Enterobacteriaceae strains permeated with $(KFF)_3K$-FAM. $(KFF)_3K$-FAM permeation was performed based on the conditions after optimization (2 μM, OT, 1 h, 50% PBS; OT: optimal growth temperature) followed by 0.1% Trypan Blue staining. Scale bar is 5 μm.
